# Role of GPR55 during Axon Growth and Target Innervation

**DOI:** 10.1523/ENEURO.0011-15.2015

**Published:** 2015-11-09

**Authors:** Hosni Cherif, Anteneh Argaw, Bruno Cécyre, Alex Bouchard, Jonathan Gagnon, Pasha Javadi, Sébastien Desgent, Ken Mackie, Jean-François Bouchard

**Affiliations:** 1 School of Optometry, University of Montreal, Montreal, Quebec H3T 1P1, Canada; 2 VIB Vesalius Research Center, KU Leuven, Leuven, Belgium, 3000; 3 CHU Sainte-Justine, Montreal, Quebec H3T 1C5, Canada; 4 Department of Physiological and Brain Sciences, Indiana University, Bloomington, Indiana 47405-7000

**Keywords:** axon guidance, development, GPR55 receptor, growth cone, retinal ganglion cell, vision

## Abstract

Guidance molecules regulate the navigation of retinal ganglion cell (RGC) projections toward targets in the visual thalamus. In this study, we demonstrate that the G-protein-coupled receptor 55 (GPR55) is expressed in the retina during development, and regulates growth cone (GC) morphology and axon growth. *In vitro*, neurons obtained from *gpr55* knock-out (*gpr55^-/-^*) mouse embryos have smaller GCs, less GC filopodia, and have a decreased outgrowth compared with *gpr55^+/+^* neurons. When *gpr55^+/+^* neurons were treated with GPR55 agonists, lysophosphatidylinositol (LPI) and O-1602, we observed a chemo-attractive effect and an increase in GC size and filopodia number. In contrast, cannabidiol (CBD) decreased the GC size and filopodia number inducing chemo-repulsion. In absence of the receptor (*gpr55^-/-^*), no pharmacologic effects of the GPR55 ligands were observed. *In vivo*, compared to their wild-type (WT) littermates, *gpr55^-/-^* mice revealed a decreased branching in the dorsal terminal nucleus (DTN) and a lower level of eye-specific segregation of retinal projections in the superior colliculus (SC) and in the dorsal lateral geniculate nucleus (dLGN). Moreover, a single intraocular injection of LPI increased branching in the DTN, whereas treatment with CBD, an antagonist of GPR55, decreased it. These results indicate that GPR55 modulates the growth rate and the targets innervation of retinal projections and highlight, for the first time, an important role of GPR55 in axon refinement during development.

## Significance Statement

The implication of a novel G-protein-coupled receptor, GPR55, in neurodevelopment allows the identification of new potential therapeutic targets for abnormal development and regeneration of the CNS.

## Introduction

The G-protein-coupled receptor 55 (GPR55) is a 319-amino acid protein that was identified, cloned, and mapped to human chromosome 2q37 in 1999 (Sawzdargo et al., 1999). GPR55 is expressed in the CNS, as well as in intestine, bone marrow, immune and endothelial cells, spleen, and platelets (Sawzdargo et al., 1999; Ryberg et al., 2007; Waldeck-Weiemair et al., 2008; Pietr et al., 2009; Balenga et al., 2011; Henstridge et al., 2011; Rowley et al., 2011). GPR55 is phylogenetically distinct from the traditional cannabinoid receptors and shows low amino acid identity compared with cannabinoid receptors 1 and 2: (CB1R, 13.5%; CB2R, 14.4%; Baker et al., 2006). Despite its activation by several cannabinoid ligands, GPR55 lacks the classical cannabinoid-binding pocket present in both CB1R and CB2R (Petitet et al., 2006; Kotsikorou et al., 2011). Therefore, GPR55 is likely a receptor for small lipid mediators and some synthetic cannabinoids and related molecules. The lipid lysophosphatidylinositol (LPI), which activates GPR55 but not CB1R or CB2R, was the first endogenous ligand identified for this receptor (Oka et al., 2007; Lauckner et al., 2008; Waldeck-Weiermair et al., 2008; Henstridge et al., 2009; Oka et al., 2009). A recent study showed that phosphatidyl-b-d-glucoside (PtdGlc), a membrane glycerophospholipid (Nagatsuka et al., 2003) and its hydrolytic derivative lyso-phosphatidyl-b-d-glucoside (LysoPtdGlc) mediate guidance of nociceptive afferent axons in the developing spinal cord via GPR55 (Guy et al., 2015). The atypical synthetic cannabinoid O-1602, with no significant binding affinity for either CB1 or CB2, also activates GPR55 and is considered as a GPR55 agonist (Johns et al., 2007; Ryberg et al., 2007; Pertwee, 2007; Waldeck-Weiermair et al., 2008; Whyte et al., 2009; Romero-Zerbo et al., 2011; Schicho et al., 2011; Sylantyev et al., 2013). Conversely, cannabidiol, a constituent of cannabis sativa and an analog of O-1602, is an effective GPR55 antagonist, with low affinity for CB1R/CB2R (Ryberg et al., 2007; Pertwee, 2007; Ross, 2009; Whyte et al., 2009; Sylantyev et al., 2013). GPR55 primarily signals via the activation of ERK1/2 and RhoA pathways, the release of calcium from intracellular stores, and the stimulation of several transcriptional factors (Ryberg et al., 2007; Lauckner et al., 2008; Henstridge et al., 2010). *gpr55* mRNA is expressed in numerous CNS-derived cells and tissues (Henstridge et al., 2011) and the receptor appears to be expressed in both neurons and glia (Pietr et al., 2009). GPR55 protein is present in mouse dorsal root ganglia (Lauckner et al., 2008), in the hippocampus (Sylantyev et al., 2013), and in the adult vervet monkey retina (Bouskila et al., 2013). Interestingly, a recent study using differentiated PC12 cells reported a role for GPR55 in neurite dynamics (Obara et al., 2011). Based on these reports, it is plausible to speculate that GPR55 plays a role during axonal navigation and refinement. Throughout development, the retinal ganglion cell (RGC) axons navigate to their thalamic (dorsal lateral geniculate nucleus; dLGN) and midbrain (superior colliculus; SC) targets to form functional synaptic connections (Erskine and Herrera, 2007). In the present study, we assessed the role played by GPR55 during axon growth and its possible implication in visual target innervation. We used the rodent neurovisual system to demonstrate a mechanism by which GPR55 influences axon growth. We found that during development, neurons express GPR55. Furthermore, *in vitro* and *in vivo* genetic and pharmacologic manipulations of GPR55 affect RGC axon growth and retinothalamic development. Importantly, we observed that the ERK1/2 and RhoA pathways are necessary for GPR55-induced effects on growth cone morphology and axon outgrowth. This study is the first demonstration that GPR55 is expressed in the developing CNS and plays an important role in axon navigation and brain wiring.

## Materials and Methods

### Animal experimentation

All animal procedures were performed in accordance with the relevant university’s animal care committee’s regulations and approval. Male and female mice and hamsters were used in this study. No statistical differences have been observed between both genders. All procedures were performed in accordance with the guidelines from the Canadian Council on Animal Care and the NIH Guide for the care and use of laboratory animals, and were approved by the ethics committee on animal research of the Université de Montréal. The *cnr1* ^-/-^ mice in which *cnr1* is deleted were obtained from Beat Lutz (Institute of Physiological Chemistry and Pathobiochemistry, University of Mainz, Germany). The cnr2 mice in which CB2 is not functional were purchased from The Jackson Laboratory. The *gpr55^-/-^* mice were acquired from the Texas Institute for Genomic Medicine. For all experiments, heterozygous females and males were mated to generate *gpr55^+/+^* and *gpr55^-/-^* littermates. Animal procedures involving *gpr55* mice were approved by the Indiana University Bloomington Institutional Animal Care and Use Committee and were conducted in compliance with the U.S. Department of Health and Human Services guidelines. All the surgical procedures were carried out under deep general anesthesia using either hypothermia (pups < P4) or isoflurane (pups >P4 and adults).

### Reagents

Bovine serum albumin (BSA), brain-derived neurotrophic factor (BDNF), ciliary neurotrophic factor (CNTF), DNase, forskolin (FSK), Hoechst 33258, insulin, laminin, monoclonal anti-β-actin, monoclonal anti-MAP Kinase (diphosphorylated ERK-1/2), poly-d-lysine, progesterone, putrescine, pyruvate, selenium, LPI from soybean, and trypsin, triiodothyronine, DEPEC, triethyl ethanol amine, prehybridization solution, formamide glutaraldehyde 50% solution were purchased from Sigma-Aldrich. CBD, Tocrifluor (T1117), and O-1602 (5-methyl-4 [(1R,6R)-3-methyl-6-(1-cyclohexen-1-yl]-1,3-benzenediol) from Tocris Bioscience. B27, N2, Dulbecco’s phosphate-buffered saline (DPBS), fetal bovine serum (FBS), glutamine, neurobasal media, penicillin-streptomycin, S-MEM, sodium pyruvate, and AlexaFluor-conjugated secondary antibodies (AlexaFluor 488 and AlexaFluor 555) were purchased from Life Technologies. The normal donkey serum (NDS), goat, and HRP coupled secondary antibodies raised against rabbit IgG (H+L) or mouse IgM (µ chain specific) were from Jackson ImmunoResearch. Rabbit-anti-mouse-macrophage was obtained from Accurate Chemical. Anti-ERK1/2 and anti-GAP-43 were acquired from EMD Millipore. Anti-RhoA and anti-phosphorylated RhoA were purchased from Santa Cruz Biotechnology. Anti-PKA, anti-phosphorylated PKA, anti-AKT and anti-phosphorylated AKT were purchased from Cell Signaling. The antibody directed against GPR55 (Kumar et al., 2012; Bouskila et al., 2013), the ROCK1 inhibitor (Y-27632) and the GPR55 blocking peptide were purchased from Cayman Chemical. ERK 1/2 inhibitor (CI-1040) was obtained from Selleck Chemicals. LNAC was acquired from EMD. Avidin-biotin-peroxidase complex ABC Kit and donkey anti-goat biotinylated secondary antibody were obtained from Vector Laboratories. The B fragment of the cholera toxin (CTb) and goat-anti-CTb were from List Biological Laboratories. Buffer kit, RnaseA buffer, and SSC buffer were from Ambion.

### Tissue preparation for immunohistochemistry

Newborn hamsters were deeply anesthetized by hypothermia, whereas adult mice were euthanized by an overdose of isoflurane. A transcardiac perfusion was conducted with phosphate-buffered 0.9% saline (PBS; 0.1m, pH 7.4), followed by phosphate-buffered 4% paraformaldehyde (PFA), until the head was fixed. The nasal part of the eyes of hamsters, mouse embryos, and adult mice was marked with a suture and removed. Two small holes were made in the cornea before a first postfixation in 4% PFA for a period of 30 min. The cornea and lens were removed and the eyecups were postfixed for 30 min in 4% PFA. The eyecups were then washed in PBS, cryoprotected in 30% sucrose overnight, embedded in Neg 50 tissue Embedding Media (Fisher Scientific), flash-frozen, and kept at −80°C. Sections (14 µm thick) were cut with a cryostat (Leica Microsystems) and placed on gelatin/chromium-coated slides.

### Immunohistochemistry

The presence of GPR55 during the early development of the mouse and hamster retinas was investigated by immunohistochemistry. Retinal sections were washed in 0.1 m PBS, postfixed for 5 min in a 70% solution of ethanol, rinsed in 0.03% Triton X-100 in buffered saline, and blocked in 10% NDS and 0.5% Triton X-100 in buffered saline for 1 h. The sections were then coincubated overnight with rabbit anti-GPR55 antibody. After incubation with the primary antibody, the sections were washed in buffered saline, blocked for 30 min, and incubated for 1 h with secondary antibody: AlexaFluor donkey anti-rabbit 488. Because of the absence of immunoreactivity of several antibodies labeling the ganglion cells during the embryonic development of the mouse retina, we used a nucleus marker (Sytox) to visualize the cell somas. After washes, the sections were mounted with a homemade PVA-Dabco mounting media.

### Fluorescent *in situ* hybridization

All solutions used for the fluorescent *in situ* hybridization experiments were prepared with RNase-free reagents and diethylpyrocarbonate (DEPC)-treated double-deionized water (ddH_2_O). Glassware and instruments were RNase-decontaminated using RNase away solution (Fisher Scientific). Probes were designed in our laboratory and made by Sigma-Aldrich. *In situ* hybridization to detect *gpr55* mRNA was performed following the instructions as described by Zangenehpour and Chaudhuri (2001). For detection of each species’ *gpr55* RNA, two specific probes were used, and all were coupled to a fluorescent dye: 6-fluorescein phosphoramidite (6-FAM). As a positive control, a poly-T probe was used. Primer sequences (5'-3') for *in situ* hybridization are as follows:

Mouse Probe 1: [6FAM]ACATGCTGATGAAGTAGAGGCA

Mouse Probe 2: [6FAM]TTGGTTCTTCTGCTTCATACA

Hamster Probe 1: [6FAM]TGAAGCAGATGGTGAAGACACT

Hamster Probe 2: [6FAM]AGTTGCAGGAACAAGCTGATGT

The mouse probes were based on the truncated sequence of nucleotides in *gpr55^-/-^* mice. Pictures showing expression patterns were taken using a Leica TCS SP2 confocal microscope (Leica Microsystems).

### Retinal explant culture

The retinas were isolated from mouse embryonic day (E)14/15 embryos, dissected into small segments in ice-cold DPBS and platted on 12 mm glass coverslips previously coated with poly-d-lysine (20 µg/ml) and laminin (5 µg/ml) in 24-well plates. The explants were cultured in neurobasal supplemented with 100 U/ml penicillin, 100 µg/ml streptomycin, 5 µg/ml LNAC, 1% B27, 40 ng/ml selenium, 16 µg/ml putrescine, 0.04 ng/ml triiodothyronine, 100 µg/ml transferrin, 60 ng/ml progesterone, 100 µg/ml BSA, 1 mm sodium pyruvate, 2 mm glutamine, 10 ng/ml CNTF, 5 μg/ml insulin, and 10 μM FSK at 37°C and 5% CO2. At 0 days *in vitro* (DIV; 1 h after plating), the explants were treated for 15 h for projection analysis or for 1 h at 1 DIV for growth cone analysis. Photomicrographs were taken using an Olympus IX71 microscope (Olympus) and analyzed with Image Pro Plus 5.1 software (Media Cybernetics). The total length of axon bundles was quantified and expressed as mean ± SEM. Statistical significance of differences between means was evaluated by ANOVA with Bonferroni’s *post hoc* test (Systat Software).

### Purified retinal ganglion cell culture

RGCs from P7–P8 mice (Charles River Laboratories) were purified and cultured according to a protocol previously described by Barres et al. (1988). In brief, following enucleation, retinas were dissected and enzymatically dissociated, at 37°C for 30 min, in a papain solution (15 U/ml in DPBS) containing 1 mm l-cysteine. The retinas were then triturated sequentially, with a 1 ml pipette, in a solution containing ovomucoid (1.5 mg/ml), DNase (0.004%), BSA (1.5 mg/ml), and rabbit antibodies directed against mouse macrophages (1:75) to yield a suspension of single cells. The suspension was centrifuged and washed in a high concentration ovomucoid-BSA solution (10 mg/ml for each in DPBS). The dissociated cells were resuspended in DPBS containing BSA (0.2 mg/ml) and insulin (5 μg/ml). RGCs were purified using the two-step panning procedure (Barres et al., 1988; Meyer-Franke et al., 1995). Briefly, to remove macrophages, the retinal suspension was incubated at room temperature in Petri dishes coated with affinity-purified goat anti-rabbit IgG (H+L). The nonadherent cells were then transferred to a Petri dish that had been coated with affinity-purified goat anti-mouse IgM (μ chain specific) followed by anti-Thy-1.2 monoclonal IgM. The adherent RGCs were first released enzymatically by incubating them in a 0.125% trypsin solution at 37°C and 5% CO2 followed by manually pipetting an enzyme inhibitor solution (30% FBS in neurobasal) along the surface of the dish. Purified RGCs were plated on poly-d-lysine- (10 μg/ml) and laminin- (5 μg/ml) coated glass coverslips (number 0 Deckgläser; Carolina Biological) in 24-well plates. RGCs were cultured in 600 μl of serum-free medium modified from Bottenstein and Sato (1979). Neurobasal media was supplemented with B27, selenium, putrescine, triiodothyronine, transferrin, progesterone, pyruvate (1 mm), glutamine (2 mm), CNTF (10 ng/ml), BDNF (50 ng/ml), insulin (5 μg/ml), and FSK (10 μm). RGCs were cultured at 37°C and 5% CO2. All experiments on purified RGCs were performed 36–40 h following plating.

### Primary neuron culture

Primary cortical neurons were used in this study because of the large amount of neurons that can easily be cultured and harvested for biochemical assays, which is not possible with RGCs. *Cnr1*, *cnr2*, and *gpr55* pregnant knock-out mice and their respective wild-type (WT) controls were used. CD1 staged pregnant mice were obtained from Charles River Laboratories. E14/15 embryo brains were dissected and the superior layer of each cortex was isolated and transferred in 2ml S-MEM at 37°C with 2.5% trypsin and 2 mg/ml DNase for 15 min. Pellet was transferred into 10 ml S-MEM with 10% FBS and stored at 4^o^C. After centrifugation, pellet was again transferred in 2 ml S-MEM supplemented with 10% FBS and triturated 3 to 4 times. The supernatant was transferred in 10 ml neurobasal medium. Dissociated cells were counted and plated at 50,000 cells per well on 12 mm glass coverslips previously coated with poly-d-lysine (20 µg/ml). Neurons were cultured for 2 d in neurobasal medium supplemented with 1% B-27, 100 U/ml penicillin, 100 µg/ml streptomycin, 0.25% N_2_, and 0.5 mm glutamine. Neurons were treated with either GPR55 agonists (1 µm LPI or 300 nm O-1602), a GPR55 antagonist (300 nm CBD), ERK 1/2 inhibitor (20 µm CI-1040), ROCK1 inhibitor (20 µm Y27632), for 60 min for growth cone (GC) morphology or 2, 5, 10 and 15 min for ERK-1/2, RhoA, AKT, and PKA protein quantification using Western blots.

### Growth cone behavior assay

Embryonic retinal explants were cultured on a coverglass in a borosilicate chamber (Lab-Tek) for 2 DIV and placed in an incubator mounted on an inverted microscope. They were kept at 37°C and 5% CO_2_ with a Live Cell chamber (Neve Bioscience) throughout the whole experiment. A microgradient was created using a Picoplus micro-injector (Harvard Apparatus). Glass micropipettes with a diameter of the tip of 2-3 µm were positioned at 45° and at 100 µm away from the GC of interest (Argaw et al., 2011; Duff et al., 2013).

### Immunocytochemistry

After treatments, retinal explants and primary cortical neuron cultures were washed with PBS, pH 7.4, fixed in 4% PFA, pH 7.4, and blocked with 2% NGS and 2% BSA in PBS containing 0.1% Tween 20, pH 7.4, for 30 min at room temperature. Neurons were then incubated overnight at 4°C in blocking solution containing anti-GAP-43 (1:1,000) for GC morphology analysis, anti-GPR55 (1:500) or Tocrifluor T1117 (3µm) for GPR55 protein expression, MAP2 (1:500) or NFM (1:500). The following day, neurons were washed and labeled with AlexaFluor secondary antibodies (488 and 555) and Hoechst 33258, and the coverslips were mounted with a homemade Dabco-PVD mounting media (Ono et al., 2001).

### Western blot analysis

Mouse embryos or pups were euthanized at various ages, namely: E14/15, E16/17, E18/19, postnatal day (P)1, and P3. Following deep anesthesia by hypothermia, eyes were immediately removed for Western blot analysis. Retinas were dissected on ice, homogenized by hand in radioimmunoprecipitation assay (RIPA) buffer (150 mm NaCl, 20 mm Tris, pH 8.0, 1% NP-40, 0.1% SDS, 1 mm EDTA). This buffer was supplemented with a protease inhibitor mixture [aprotinin, leupeptin, pepstatin (1 μg/ml) and phenylmethylsulfonyl fluoride (0.2 mg/ml); Roche Applied Science]. Samples were then centrifuged at 13,000 rpm at 4°C for 10 min and supernatants were removed and stored. Protein content was equalized using BCA Protein Assay kit (Thermo Scientific). In another set of experiments, primary cortical neurons were cultured for 2 DIV at a density of ≈250,000 cells/dish in 35 mm poly-d-lysine coated petri dishes. Following treatment, neurons were washed once with ice-cold PBS, pH 7.4, and then lysed with Laemmli sample buffer. Primary antibodies were used at the following concentrations: anti-GPR55 (1:500), anti-β-actin (1:5,000), anti-AKT (1:1,000), anti-p-AKT (1:1,000), anti-ERK1/2 (1:5,000), anti-p-ERK1/2 (1:2,000), anti-RhoA (1:1,000), and anti-p-RhoA (1:1,000). Results were visualized using homemade enhanced chemiluminescent Western blot detection reagents (final concentrations: 2.5 mm luminol, 0.4 mm p-coumaric acid, 0.1 m Tris–HCl, pH 8.5, 0.018% H_2_O_2_).

### Intraocular injections

Syrian golden hamsters (Charles River Laboratories) are born with a premature visual nervous system (Clancy et al., 2001). These mammals were used for studies investigating the implication of GPR55 ligands during retinal ganglion cell projection growth during development *in vivo*. Twenty-four hours following birth, at P1, anesthetized hamsters received a unilateral intraocular injection of 2 µl solution of CTb, with either 0.9% saline solution, 1 mm of LPI or 300 µm of CBD. Briefly, to access to the right eye, a small incision was made in the eyelids under an operating microscope. A glass micropipette attached to a 10 µl Hamilton syringe was used for the injection. Insertion of the micropipette into the vitreous was conducted carefully at an angle to avoid damage to the lens. Following the injection, we closed the eyelids using surgical glue (Vetbond, 3M). The same surgical procedures were performed using P1 and adult *gpr55^+/+^* and *gpr55^-/-^* mice to allow the detection of any morphologic or growth difference between the genotypes. For eye-specific segregation studies in the dLGN, *gpr55^-/-^* and *gpr55^+/+^* adult mice received an intraocular injection of CTb conjugated to AlexaFluor 555 into the left eye and CTb coupled to AlexaFluor 488 into the right eye (2 μl; 0.5% in sterile saline). Two or 4 d after the injection for mice (pups and adults) and hamsters, respectively, the animals were anesthetized and perfused transcardially with 0.1 m PBS, pH 7.4, followed by 4% PFA in PBS. The brains were removed, postfixed overnight at 4ºC and cryoprotected by infiltration of buffered sucrose. Then, brains were frozen and kept at −80ºC.

The effects of the intraocular injection of GPR55 agonist and antagonist were visualized by immunohistochemistry according to a protocol previously described in (Argaw et al., 2008). Briefly, 40 µm thick coronal sections of tissue were incubated in 90% methanol and 0.3% H_2_O_2_ in 0.1 m PBS, pH 7.4, for 20 min. They were then rinsed and incubated in 0.1 m glycine/PBS for 30 min, followed by an overnight incubation (4°C) in PBS containing 4% NDS, 2.5% BSA, and 1% Triton X-100. The sections were subsequently rinsed and immersed for 48 h at room temperature in a solution containing goat anti-CTb diluted 1:4,000 in PBS with 2% NDS, 2.5% BSA, and 2% Triton X-100. Afterward, the sections were rinsed and incubated in 2% NDS and 2.5% BSA/PBS for 10 min. This was followed by a 1 h incubation in donkey anti-goat biotinylated secondary antibody diluted 1:200 in PBS with 2% NDS, 2.5% BSA, and 1% Triton X-100. Tissue was rinsed, incubated in 2% NDS and 2.5% BSA in PBS for 10 min, and subsequently processed by an avidin-biotin-peroxidase complex ABC Kit (diluted 1:100 in PBS) for 1 h in the dark at room temperature. The sections were then rinsed and preincubated in 3, 3′-diaminobenzidine tetrahydrochloride (DAB) in PBS for 5 min. The peroxidase reaction product was visualized by adding 0.004% H_2_O_2_ to the DAB solution for 2–4 min. Sections were finally washed five times (1 min each) with PBS, mounted on gelatin-chromium alum-subbed slides, air-dried, dehydrated in ethanol, cleared in xylenes, and coverslipped with Depex (EMS).

### Quantification method

Photomicrographs were taken with an inverted Olympus IX71 microscope (Olympus) and an Evolution VF camera (MediaCybernetics). The images were quantified using Image Pro Plus 5.1 image analysis software. The growth of axon branches was quantified on consecutive photomicrographs of coronal slices of brain tissue comprising the DTN. On each photomicrograph, the distance between the lateral border of the nucleus of interest and the tips of the longest axon branches was measured. To take into account for differences in brain sizes, axon branch lengths were normalized with the interthalamic distance (distance between the right and left lateral borders of the thalamus). Axon collateral number was quantified on consecutive photomicrographs comprising the DTN using an adaptation of the Sholl technique as described by Duff et al. (2013). Values are expressed as the mean ± SEM. Statistical significance of differences between means was evaluated by ANOVA with Bonferroni’s *post hoc* test (Systat).

For eye-specific segregation quantification in the dLGN, images were collected and measured by an observer “blind” to the experimental conditions to minimize any bias. Universal gains and exposures were established for each label. Raw images of the dLGN were imported to MATLAB and an area of interest comprising the dLGN was cropped excluding the ventral lateral geniculate nucleus and the intergeniculate leaflet, then the degree of left and right eye projection overlap was quantified using an established multi-threshold method of analysis (Torborg and Feller, 2004; Bjartmar et al., 2006; Stevens et al., 2007). This approach allows for a better analysis of overlapping regions independent of the threshold. Values are expressed as the mean ± SEM. Significance of differences between means was evaluated by student *t* test analysis (Systat).

### Genotyping

Animals were genotyped as described by Wu et al. (2010). Tail samples were immersed in 50 mm NaOH, boiled for 30 min, vortexed vigorously for 10 s, and neutralized with 1 m Tris-HCl, pH8.0. Tail lysates obtained were vortexed again for 10 s and centrifuged at 16,100 × *g* for 1 min. PCR reactions were conducted with a mixture of two primer pairs to generate the following amplicons: the 441 bp for the WT *gpr55* allele and the 301 bp for the neo allele. The primer sequence was for the WT allele: 5′-GCCATCCAGTACCCGATCC-3′ and 5′-GTCCAAGATAAAGCGGTTCC-3′ and for the *gpr55* mutant allele the sequence: 5′-GCAGCGCATCGCCTTCTATC-3′ and 5′-TCAAGCTACGTTTTGGGTT-3′. The PCR cycle conditions were: 5 min at 95°C, 36 cycles of three steps (50 s at 94°C, 40 s at 55°C, and 40 s at 72°C), then 5 min at 72°C using the standard PCR reagents. A similar genotyping protocol was performed on mouse genomic tail DNA using sense primers: 5′-GCTGTCTCTGGTCCTCTTAAA-3′; 5′-GGTGTCACCTCTGAAAACAGA-3′ for the WT allele and 5′-CCTACCCGGTAGAATTAGCTT -3′ to detect the *Cnr1^-/-^* allele. The primer sequences for the *Cnr2* WT allele were 5′-GGAGTTCAACCCCATGAAGGAGTAC-3′ and

5′-GACTAGAGCTTTGTAGGTAGGCGGG-3′ and for the *Cnr2* mutant allele, the sequence was 5′-GGGGATCGATCCGTCCTGTAAGTCT-3′.

## Results

### GPR55 expression in the developing retina

**Figure 1.**
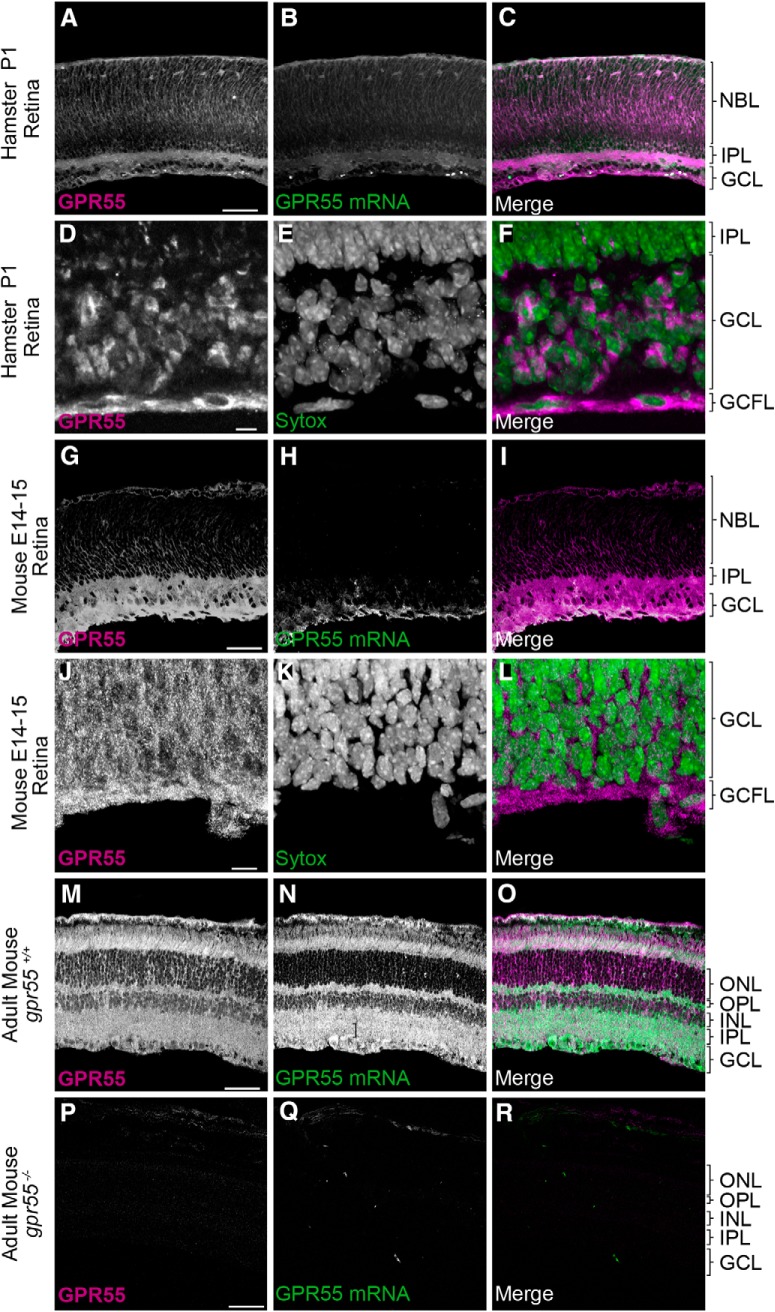
**GPR55 protein and mRNA expression in the retina. *A*–*F***, At P1, GPR55 protein and mRNA are expressed in the hamster retina (***A*–*C***), expression of GPR55 protein in the ganglion cell layer (***D***–***F***). ***G***–***L***,GPR55 protein and mRNA are present in the E14/15 mouse retina (***G*–*I***), especially for GPR55 protein in the ganglion cell layer (***J***–***L***). ***M***–***O***, GPR55 protein and mRNA are expressed in the adult mouse retina. ***P***–***R***, The specificity of the antibody and the mRNA probe was validated using *gpr55^-/-^ mice*. Scale bars: ***A***–***C***, ***M***–***R***, 75 μm; ***G***–***I***, 30 μm; ***D***–***F***, ***J***–***L***, 25 μm. NBL, Neuroblast layer; IPL, inner plexiform layer; INL, inner nuclear layer; OPL, outer plexiform layer; ONL, outer nuclear layer; GCL, ganglion cell layer; GCFL, ganglion cell fiber layer.

We used hamster and mouse retinas to evaluate the presence of GPR55 and its possible involvement during retinal projection navigation. Both GPR55 protein and mRNA were expressed in the hamster retina. At P1, GPR55 protein was present in the ganglion cell (GCL), ganglion cell fibers (GCFLs), inner plexiform (IPL) and neuroblast (NBL) layers, whereas GPR55 mRNA was present in the GCL (Fig. 1*A*–*F*). GPR55 protein was also expressed in the GCL, GCFL, IPL, and NBL, whereas GPR55 mRNA was localized in GCL in retina of E14/15 mouse embryos (Fig. 1*G*–*L*). GPR55 protein and mRNA were both detected in the adult *gpr*55^+/+^ mouse retina (Fig. 1*M*–*O*) but not in the *gpr*55^-/-^ retina (Fig. 1*P*–*R*).

**Figure 2.**
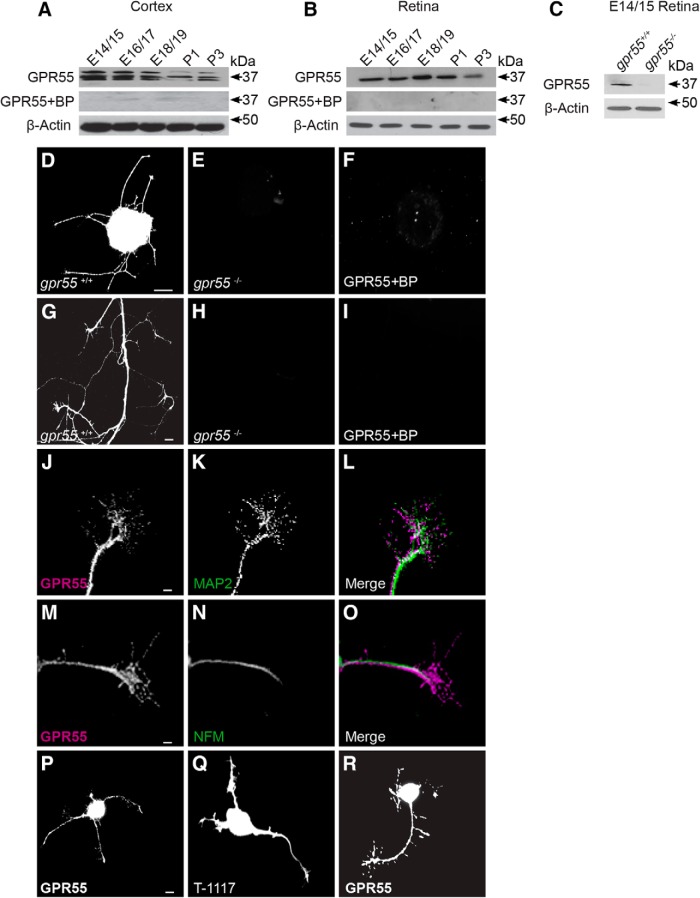
**GPR55 protein expression in retinal explants and primary cortical neurons. *A*,** Expression of GPR55 in the mouse cortex and **(*B*)** retina at different developmental stages. ***C***, Expression of GPR55 in the retina of *gpr55^+/+^* and *gpr55^-/-^* mouse embryo (E14/15). ***D***–***F***, Expression of GPR55 in retinal explants from *gpr55^+/+^* and *gpr55^-/-^* mice, and in the presence of the specific blocking peptide (BP). ***G***–***I***, E14/15 axons and growth cones of retinal explants from *gpr55^+/+^* and *gpr55^-/-^* mice, and in the presence of the specific BP. ***J***–***L***, The expression of GPR55 in dendrites (MAP2) and (***M***–***O***) axons (NFM) of RGCs. ***P***, Expression of GPR55 in primary cortical neuron using GPR55 antibody, (***Q***) using 3 µm of specific GPR55 fluorescent Tocrifluor ligand T-1117 and (***R***) using GPR55 antibody in a purified RGC culture. Scale bars: ***D***–***F***, 100 μm; ***G***–***I***, ***P***–***R***, 10 μm; ***J***–***O***. 5 μm.

Using Western blot, GPR55 protein was detected in the brain and retina of mouse embryos and pups from E14/15 to P3. The signal was abolished in the presence of the blocking peptide for the antibody (Fig. 2*A*,*B*). The same antibody failed to detect GPR55 in retina homogenate obtained from *gpr*55^-/-^ mouse embryos (E14/15; Fig. 2*C*). In E14/15 mice retinal explants, GPR55 was present in the neurites; their GCs and filopodia (Fig. 2*D*). Explants obtained from *gpr*55^-/-^ embryos did not express GPR55 (Fig. 2*E*). Furthermore, GPR55 was not detectable after co-incubation of the antibody with its blocking peptide (Fig. 2*F*). GPR55 was present in GCs and neurites of retinal explants from *gpr*55^+/+^ (Fig. 2*G**)*. GPR55 immunoreactivity was not detectable in retinal explants obtained from *gpr*55^-/-^ mice (Fig. 2*H*) or in the presence of blocking peptide (Fig. 2*I*). In WT mice, GPR55 was expressed in dendrites (Fig. 2*J*–*L*) and axons (Fig. 2*M*–*O*). It was also present in cortical neuron somas, neurites, and GCs (Fig. 2*P*). Moreover, the expression of GPR55 was investigated using Tocrifluor (T-1117), a fluorescent ligand of GPR55 (Sylantyev et al., 2013), confirming the presence of GPR55 in primary cortical neurons (Fig. 2*Q*). Isolated retinal ganglion cells from E14/15 mice expressed GPR55 in their GCs and filopodia (Fig. 2*R*). Overall, we observed that during development, GPR55 was present in the retinas of mouse and hamster.

### GPR55 ligands reorganize GC morphology and modulate axon growth

**Figure 3.**
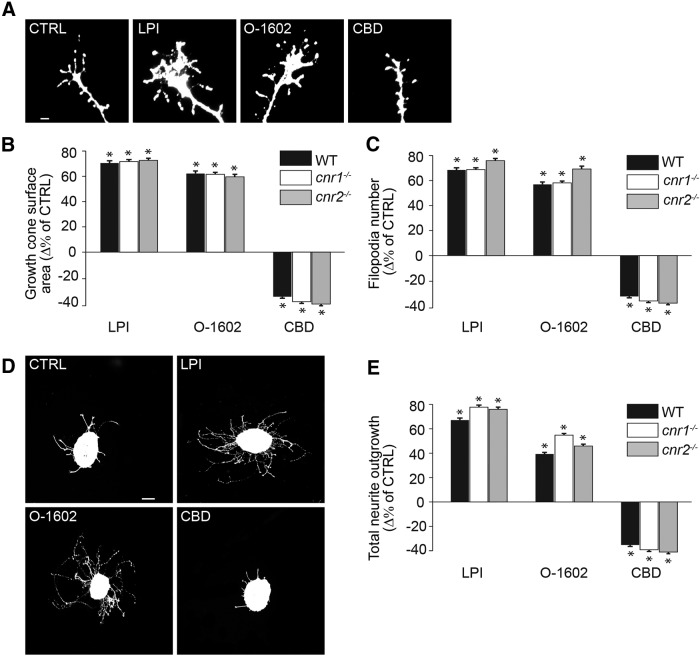
**GPR55 ligands reorganize the morphology of the GC and modulate axon growth via a cannabinoid independent pathway. *A***–***C***, Growth cone surface area and filopodia number of retinal projection GCs after a 60 min treatment with GPR55 agonists LPI (1 µm) and O-1602 (300 nm) or antagonist CBD (300 nm) in WT, *cnr1^-/-^*, and *cnr2^-/-^* mice. ***D***, ***E***, Total retinal neurite growth of retinal explants cultured for 1 DIV and treated for 15 h with LPI (1 µm), O-1602 (300 nm), and CBD (300 nm) in WT, *cnr1^-/-^*, and *cnr2^-/-^* mice. Scale bars: ***A***, 5 μm; ***D***, 100 μm. Values are presented as mean ± SEM. *Indicates significant changes between LPI, O-1602, or CBD compared with control in ***B***, ***C***, and ***E***; *p* < 0.004.

To assess the role of GPR55 during retinal axon growth and guidance, retinal explants isolated from embryonic mice were cultured for 2 DIV, and treated with pharmacologic modulators of GPR55. When retinal explants were exposed to 1µm LPI (*n* = 1005 GCs; *p < 0.0001^a^ ; Table 1) or 300 nm O-1602 (*n* = 1022 GCs; *p < 0.0001^a^) for 60 min, the GC surface area and the number of filopodia increased significantly compared with the control (*n* = 1023 GCs). In contrast, application of 300 nm CBD (*n* = 1134 GCs; *p < 0.0001^a^) to the cultures decreased the GC surface and filopodia number in RGCs neurons (Fig. 3*A*–*C*). To investigate the effects of GPR55 ligands on axon growth, retinal explants were treated for 15 h with LPI, O-1602, or CBD. Treatments with agonists 1 µm LPI (*n* = 605 explants; *p < 0.0001^b^) and 300 nm O-1602 (*n* = 595 explants; *p < 0.0001^b^) increased the total neurite growth, whereas the 300 nm CBD (*n* = 602 explants; *p < 0.0001^b^) decreased it compared with control (*n* = 720 explants; Fig. 3*D*,*E*). Interestingly, the LPI, O-1602, and CBD had similar effects in tissues obtained from WT, cannabinoid receptor 1 knock-out (*cnr1^-/-^*) or cannabinoid receptor 2 knock-out (*cnr2^-/-^*) mice (GC area, *p* = 0.37^c^; filopodia number, *p* = 0.48^c^; total neurite outgrowth, *p* = 0.29^c^; Fig. 3*B*,*C*,*E*). Together, these results indicate that ligands engaging GPR55 modulate GC morphology and axon growth in retinal explants, and that their effects are not mediated by CB1R or CB2R.

**Table 1.** Statistical Table

	**Data structure**	**Type of test**	**Power**
All statistical tests ^a–t^	Normally distributed	ANOVA with *post hoc* Bonferroni	0.9–1.0
Test ^u^	Normally distributed	Student’s *t* test	0.921

Statistical analyses were performed by ANOVA with Bonferroni’s *post hoc* test and Student’s *t* tests (Systat software).

**Figure 4.**
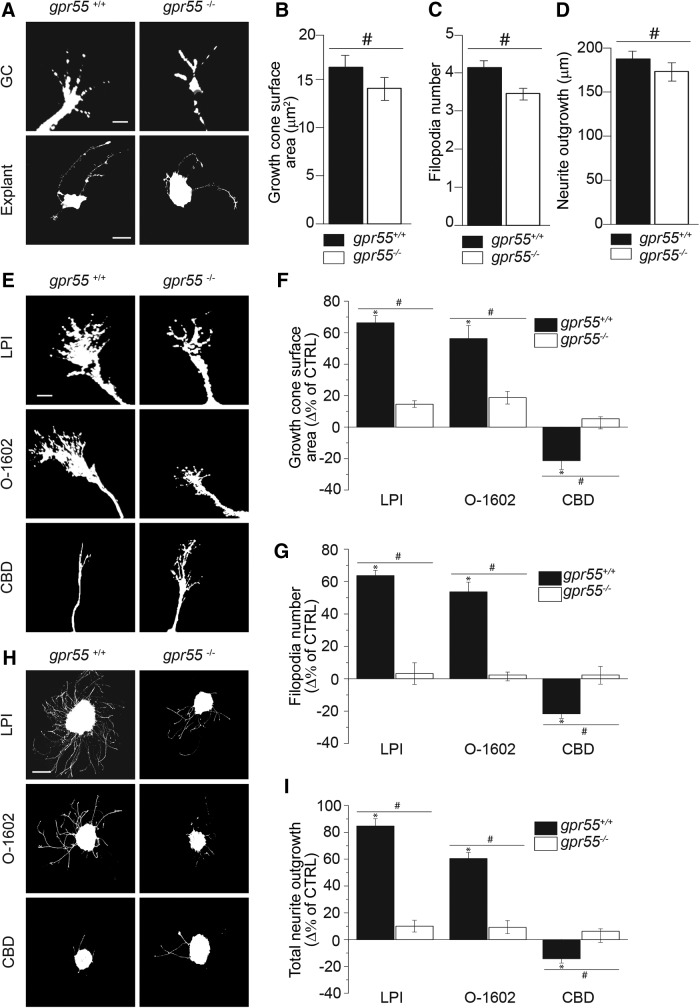
**GPR55 mediates the reorganization of the GC morphology and the modulation of axon growth. *A***–***D*,** Basal growth cone surface area, filopodia number and total neurite outgrowth in *gpr55^+/+^* and *gpr55^-/-^* retinal explants. ***E***–***G***, Growth cone surface area and filopodia number in *gpr55^+/+^* and *gpr55^-/-^* retinal explants treated for 1 h with LPI (1 µm), O-1602 (300 nm), or CBD (300 nm). ***H***, ***I***, Total neurite outgrowth in *gpr55^+/+^* and *gpr55^-/-^* retinal explant treated for 15 h with LPI, O-1602, or CBD at the same previously cited concentrations. Scale bars: ***A***, ***E***, 5μm for GC; ***A***, ***H***, 100 μm for explants. Values are presented as mean ± SEM. *Indicates a significant change induced by LPI, O-1602, or CBD compared with the control in ***F***, ***G***, and ***I***; *p* < 0.0001; #Indicates a significant change between LPI, O-1602, or CBD and the control in *gpr55^+/+^* compared to *gpr55^-/-^* in ***F***, ***G***, ***I***, and ***B***–***D***; *p* < 0.004.

To investigate the possible effect of the deletion of *gpr55*, retinal explants from E14/15 *gpr55^-/-^* embryos were cultured and compared to the ones obtained from *gpr55^+/+^* mice. The absence of GPR55 was accompanied by a significant decrease in growth cone surface area (Fig. 4*A*,*B*), filopodia number (Fig. 4*A*,*C*; *n* = 616 GCs for WT control; *n* = 218 GCs for KO; #*p* = 0.003^e^) and in total neurite outgrowth compared with WT, (*n* = 298 explants for WT control; *n* = 191 explants for *gpr*55^-/-^ control group; #*p* = 0.0001^d^; Fig. 4*A*,*D*). To confirm the involvement of GPR55 in the changes of GC morphology and retinal projection growth following treatment with LPI, O-1602 and CBD, retinal explants obtained from *gpr*55^+/+^ and *gpr*55^-/-^ mouse embryos were treated with the aforementioned agonists and antagonist. In cultures prepared from *gpr*55^+/+^ embryos, LPI (1 µm; *n* = 585 GCs; *p < 0.0001^d^) and O-1602 (300 nm; *n* = 501 GCs; *p < 0.0001^d^) increased the GC surface area and filopodia number, whereas CBD (300 nm; *n* = 547 GCs; *p < 0.0001^d^) decreased them (*n* = 616 GCs for WT control; Fig. 4*E*–*G*). These effects were absent in retinal explants obtained from *gpr*55^-/-^ embryos (LPI: *n* = 135 GCs; #*p* = 0.0035^e^; O-1602: *n* = 111 GCs; #*p* = 0.0031^e^; CBD: *n* = 167 GCs; #*p* = 0.0029^e^ compared with WT). Furthermore, the increase in total projection length after treatment with LPI (CTRL: *n* = 298 explants; LPI: *n* = 265 explants; *p < 0.0001^d^) and O-1602 (*n* = 248 explants; *p < 0.0001^d^) and the decrease induced by CBD (*n* = 273 explants; *p < 0.0001^d^) in *gpr*55^+/+^ animals were absent in the *gpr*55^-/-^ group (CTRL: *n* = 191 explants; LPI: *n* = 155 explants; #*p* = 0.0037^e^; O-1602: *n* = 108 explants; #*p* = 0.0029^e^; CBD: *n* = 127 explants; #*p* = 0.0032^e^ compared with *gpr*55^+/+^; Fig. 4*H*,*I*). These results confirm that the effects observed on GC morphology and retinal projection growth following treatments with LPI, O-1602, and CBD are mediated by GPR55. Together, these observations demonstrate that GPR55 modulates GC morphology and increases retinal projection growth.

### At low concentrations, GPR55 agonists modulate GC morphology and axon growth via the ERK1/2 pathway

**Figure 5.**
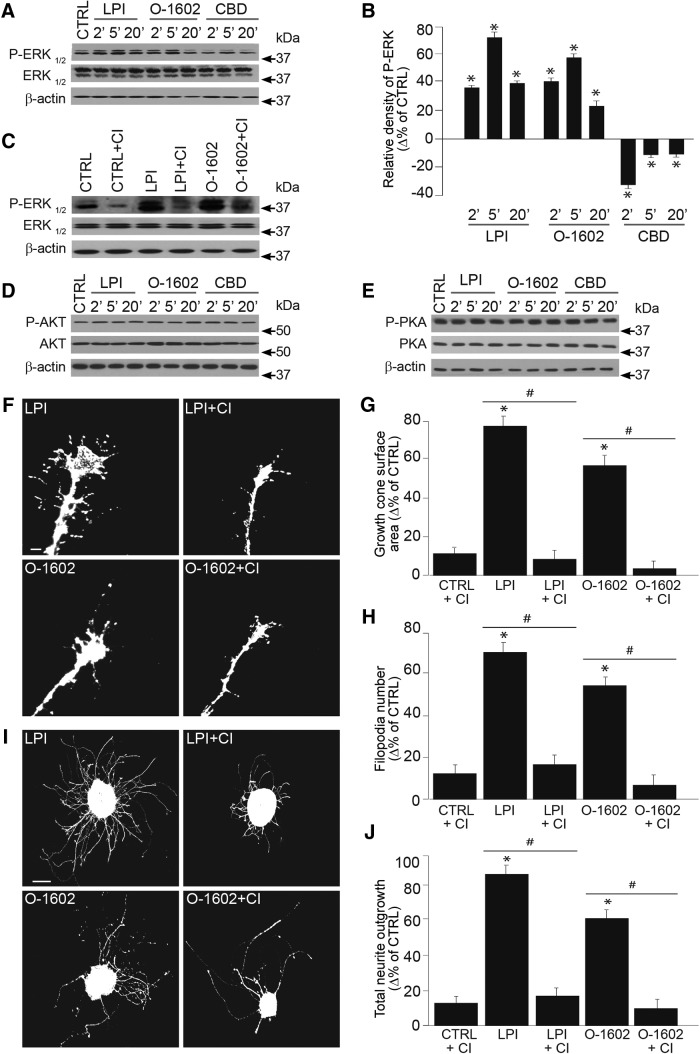
**At low concentration, GPR55 ligand modulates GC morphology and axon growth via the ERK1/2 pathway. *A***, Expression of P-ERK-1/2, ERK-1/2 and β-actin in primary cortical neurons incubated with one of the following: 1 µm LPI, 300 nm O-1602, or 300 nm CBD at 37°C for 2, 5, and 20 min. The antibody β-actin was used to verify (and correct for) equal loading in all lanes. ***B***, Histogram illustrating the quantification of ERK-phosphorylation. ***C***, ERK phosphorylation state following 15 min pretreatment with CI-1040, an ERK1/2 inhibitor, before the incubation with or without 1 µm LPI, 300 nm O-1602 or 300 nm CBD. ***D***, ***E***, AKT and PKA phosphorylation states, no significant variations were observed in the presence of GPR55 ligands for the times indicated. ***F***–***H***, Growth cone surface area and filopodia number of retinal explant treated with GPR55 agonists in the presence or the absence of the ERK inhibitor. ***I***–***J***, Total projection growth of retinal explant cultures treated with GPR55 agonists LPI and O-1602 in the presence or the absence of CI-1040. Scale bars: ***F***, 10 μm; ***I***, 100 μm. Values are presented as mean ± SEM. *Indicates a significant change compared with the control group in ***B***, ***G***, ***H***, and ***J***; *p* < 0.0001. #Indicates a significant change induced by the ERK inhibitor in ***G***, ***H***, and ***J***; *p* < 0.004.

Because it is well documented that stimulation of GPR55 and subsequently Gα13 activate ERK1/2 (Henstridge et al., 2011), we tested whether this receptor modulates the ERK1/2 pathway during axon growth and guidance. ERK1/2 phosphorylation was significantly increased following 1 µm LPI and 300 nm O-1602 stimulation, whereas 300 nm CBD application decreased it (CTRL: *n* = 15 samples; LPI*: n* = 15 samples, *p < 0.0001^f^; O-1602: *n* = 15 samples, *p < 0.0001^f^; CBD: *n* = 15 samples, *p < 0.0001^f^; Fig. 5*A*,*B*). CI-1040 (1 µm), a selective ERK1/2 inhibitor blocked the effects of LPI (1 µm) and O-1602 (300 nm) on the ERK phosphorylation (Fig. 5*C*). In primary neuronal cultures, 2, 5, and 20 min modulation of GPR55 with LPI (1 µm), O-1602 (300 nm), and CBD (300 nm) did not induce any significant changes in protein kinase B (AKT) or protein kinase A (PKA) phosphorylation levels (Fig. 5*D*,*E*). To assess the role of the ERK 1/2 pathway in GPR55 effects, retinal explants were first treated with ERK-selective inhibitor. Followed by pharmacologic activation of GPR55, CI-1040 blocked LPI (1 µm) and O-1602 (300 nm) induced increases in GC surface area and filopodia number (CTRL: *n* = 520 GCs; LPI: *n* = 517 GCs, *p < 0.0001^g^; O-1602: *n* = 509 GCs, *p < 0.0001^g^; LPI+CI: *n* = 500 GCs, #*p* < 0.0001^h^; O-1602+CI: *n* = 495 GCs; #*p* < 0.0001^h^; Fig. 5*F*–*H*). Of note, no significant difference was observed between the CTRL condition and CTRL+CI in the GC surface area and the filopodia number (CTRL: *n* = 520 GCs; CTRL+CI: *n* = 498 GCs, *p* = 0.12^g^; Fig. 5*G*,*H*). Moreover, inhibition of ERK1/2 blocked the effect of LPI and O-1602 on total projection length (CTRL: *n* = 220 explants; LPI: *n* = 215 explants, *p < 0.0001^i^; O-1602: *n* = 209 explants, *p < 0.0001^i^; LPI+CI: *n* = 210 explants, #*p* = 0.0012^j^; O-1602+CI: *n* = 200 explants, #*p* = 0.003^j^). The ERK inhibitor had no significant effect on the total projection length by itself (CTRL: *n* = 220 explants; CTRL+CI: *n* = 204 explants, *p* = 0. 2^i^; Fig. 5*I*,*J*). Together, these data demonstrate that the activation of GPR55 modulates GC morphology and axon outgrowth via the ERK1/2 pathway.

### At a higher concentration, LPI activates RhoA kinase

**Figure 6.**
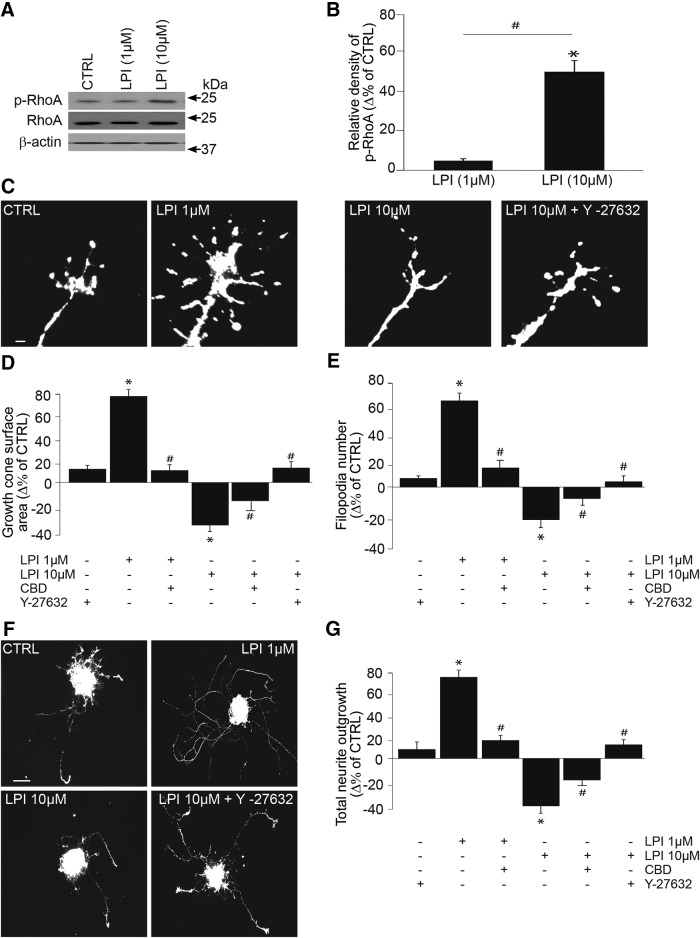
**At a higher concentration, LPI activates RhoA pathway and produces growth cone collapse and neurite repulsion. *A***, ***B***, Using p-RhoA and RhoA antibodies, RhoA activation was determined analyzing the respective phosphorylation state by Western blotting following treatment with 1 or 10 µm LPI. ***C***–***E***, Growth cone surface area and filopodia number of retinal explants after treatment with GPR55 agonist in the presence or the absence of the ROCK1 inhibitor, Y-27632. ***F***, ***G***, Total projection length of retinal explant treated with GPR55 agonist LPI (1 or 10 µm) in the presence or the absence of the Y-27632. Scale bars: ***C***, 5 μm; ***F***, 100 μm. Values are presented as mean ± SEM. *indicates a significant change compared to the control group; #indicates a significant change induced by CBD (300 nm) or Y-27632 (20 µm) in ***D***, ***E***, and ***G***; *p* < 0.0001 and *p* < 0.001 in ***B***.

In addition to ERK1/2, other signaling pathways such as RhoA, cdc42, and rac1 can be activated by GPR55 (Ryberg et al., 2007; Lauckner et al., 2008; Henstridge et al., 2010 and Obara et al., 2011). LPI at a concentration of 10 µm but not 1 µm induced an increase in RhoA phosphorylation (CTRL: *n* = 8 samples; 1 µm LPI: *n* = 8 samples, *p* = 0.31^k^; 10 µm LPI: *n* = 8 samples, *p < 0.001^k^ and #*p* < 0.001^k^ compared to 1 µm LPI; Fig. 6*A*,*B*). Interestingly, 10 µm LPI decreased the GC area, the number of filopodia (CTRL: *n* = 560 GCs; LPI 10 µm: *n* = 547 GCs, *p < 0.0001^l^) and the total projection length compared with the control (CTRL: *n* = 260 explants; LPI 10 µm: *n* = 217 explants, *p < 0.0001^m^), whereas 1 µm LPI increased them (CTRL: *n* = 560 GCs; LPI 1 µm: *n* = 522 GCs, *p < 0.0001^m^ and CTRL: *n* = 260 explants; LPI 1 µm: *n* = 213 explants, *p < 0.0001^m^). In the presence of CBD (300 nm), the effect of 1 µm LPI is blocked (1 µm LPI: *n* = 522 GCs; 1µm LPI +CBD: *n* = 500 GCs, #*p* < 0.0001^n^; 1 µm LPI: *n* = 213 explants; 1 µm LPI + CBD: *n* = 203 explants, #*p* < 0.0001^n^), whereas it is partially abolished for 10 µm LPI (10 µm LPI: *n* = 547 GCs; 10 µm LPI + CBD: *n* = 498 GCs, #*p* < 0.0001^n^; 10 µm LPI: *n* = 217 explants; 10 µm LPI + CBD: *n* = 218 explants, #*p* < 0.0001^n^; Fig. 6*C*–*G*). To assess whether RhoA/ROCK1 participated in the effects induced by high concentration of LPI, retinal explants were pretreated with Y-27632 (20 µm), a selective rho-associated, coiled-coil-containing protein kinase 1 (ROCK1) inhibitor. Y-27632 itself did not cause any changes in GC morphology (CTRL: *n* = 560 GCs; CTRL+ Y-27632: *n* = 480 GCs, *p* = 0.31^l^; Fig. 6*D*,*E*) or projection length (CTRL: *n* = 260 explants; CTRL+ Y-27632: *n* = 208 explants, *p* = 0.22^m^; Fig. 6*G*). ROCK1 inhibition blocked 10 µm LPI induced decreases in GC area, filopodia number and projection length (10 µm LPI: *n* = 547 GCs; 10 µm LPI +Y-27632: *n* = 488 GCs, #*p* < 0.0001^n^; 10 µm LPI: *n* = 217 explants, 10 µm LPI +Y-27632: *n* = 208 explants, #p < 0.0001^n^; Fig. 6*C*–*G*). Similar activation of RhoA after stimulation of GPR55 with its ligand LysoPtdGlc was reported during the guidance modulation of nociceptive axon projections in the developing spinal cord (Guy et al., 2015). Together, these data demonstrate that a low concentration (1 µm) of LPI activates the ERK1/2 pathway, whereas a higher concentration (10 µm) activates RhoA. This could in part explain the considerable variation in experimental results obtained by different laboratories examining GPR55 signaling (Henstridge, 2012).

### Pharmacologic manipulation of GPR55 affects RGC turning

**Figure 7.**
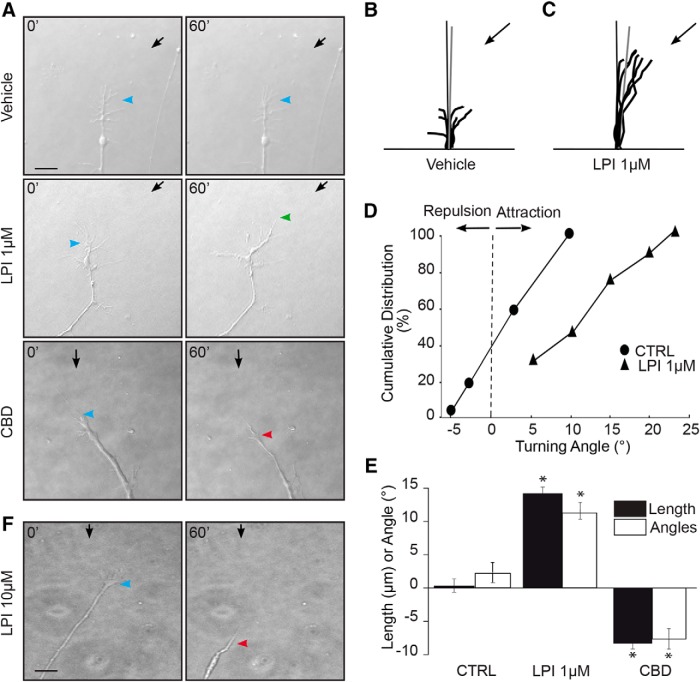
**Pharmacological modulation of GPR55 affects RGC turning *in vitro. A***, Photomicrographs of time-lapse microscopy from 1 DIV mouse retinal explant growth cone taken at *t* = 0 min and *t* = 60 min during GC turning assay experiments. Black arrows indicate the direction of the microgradient, whereas blue arrowheads indicate initial GC position. Green arrowheads show the GC position following neurite attraction and red arrowheads indicate the GC position after repulsion. ***B***, ***C***, Superimposed RGC axon trajectories over the 60 min observation period for vehicle and LPI; no significant changes were observed on growth cone behavior in the presence of the vehicle, whereas LPI increased axon growth and turning toward the pipette tip. Black arrows indicate the direction of the gradient. ***D***, Turning angle cumulative frequency curves of RGC growth cones. The turning angle of each growth cone was plotted against the percentage of growth cones turning that angle or less. ***E***, Quantification of neurite elongation and GC turning responses following drug stimulation. ***F***, Representative photomicrograph of the effect of repulsion and GC collapse created during 60 min stimulation with 10 µm LPI. Scale bars: ***A***, ***F***, 40 μm. Values are presented as means ± SEM; *indicates significant change compared with the vehicle in ***E***; p < 0.0001.

To evaluate the involvement of GPR55 in axon steering, time-lapse microscopy at 1 DIV on embryonic mouse retinal explant growth cones was performed. Arrows and arrowheads show micropipette and growth cone position, respectively. A microgradient application of 1 µm LPI elicited attractive turning, whereas 300 nm CBD induced GC collapse and neurite retraction (vehicle: *n* = 7, 1 µm; LPI: *n* = 9300 nm; CBD: *n* = 11 *p < 0.0001^°^ for length and *p < 0.0001^p^ for angles; Fig. 7*A*–*E*). The vehicle did not induce any significant directional GC turning. Interestingly, at a concentration of 10 µm, LPI induced growth cone collapse and retraction of the retinal axon (Fig. 7*F*). These data show that GPR55 can modulate axon growth and steering, and its agonist LPI can act as a chemoattractive or chemorepulsive signal depending on its concentration.

### GPR55 plays an important role during retinal projection growth and target innervation

**Figure 8.**
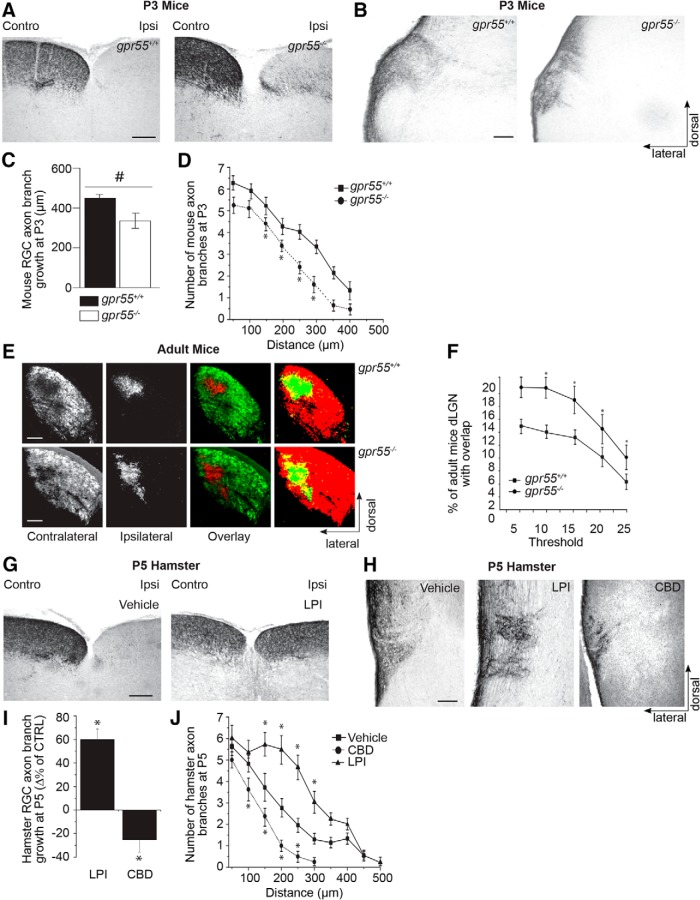
**GPR55 plays an important role during retinal projection growth and target selection *in vivo. A***, Photomicrographs of retinal projections in the SC of P3 *gpr55^+/+^* and *gpr55^-/-^* mouse pups injected, at P1, in one eye with CTb. ***B***, Photomicrographs of retinal projections in the DTN of P3 *gpr55^+/+^* and *gpr55^-/-^* mice ***C***, Quantification of retinal projection development in the DTN of *gpr55^+/+^* and *gpr55^-/-^*; collateral projection length are expressed as mean ± SEM. ***D***, Number of collateral axon branches decreases in *gpr55^-/-^* compared to *gpr55^+/+^* mice. ***E***, Images of retinogeniculate projection patterns visualized following CTb conjugated to AlexaFluor 555 (CTb-555; red) and CTb-488 (green) injections into left and right eyes of *gpr55^+/+^* and *gpr55^-/-^* adult mice. Merged images show all projections from both eyes to the dorsal lateral geniculate nucleus, overlaying projections are shown in yellow. ***F***, Quantification in *gpr55^+/+^* and *gpr55^-/-^* adult mice of the percentage of the dLGN receiving overlapping inputs as mean ± SEM. ***G***, Photomicrographs of retinal projections in the SC of P5 hamsters injected, at P1, in one eye with CTb and LPI or vehicle. A single injection of LPI induced aberrant projections in the ipsilateral SC. ***H***, Photomicrographs of P5 hamster retinal projections in the DTN in the control, LPI, and CBD groups. ***I***, Quantification of retinal projection development in the DTN; collateral projection length are expressed as mean ± SEM. ***J***, Number of collateral axon branches in treated groups compared to the control group. LPI increased axon growth and collateral branch number, whereas CBD decreased these endpoints compared with the control. Scale bars: ***A***, ***B***, ***E***, ***G***, 200 μm; ***H***, 100 μm. *n* = 8 brains per condition for P3 mice, *n* = 7 brains per condition for adult mice, and *n* = 5 brains per condition for P5 hamsters; *indicates significant change compared with the control group in ***C***, ***D***, ***F***, ***I***, and ***J***; *p* = 0.0001.

To investigate the potential role played by GPR55 during development *in vivo*, we first performed a phenotypical screening on early postnatal *gpr55^+/+^* and *gpr55^-/-^* mice to detect any morphologic differences. In P3 *gpr55^-/-^* mice, the absence of GPR55 induced a few aberrant projections in the ipsilateral side of the SC (Fig. 8*A*). Compared with the WT group, P3 *gpr55* ^-/-^ mice showed a significant decrease in RGC axon branch growth and number in the DTN (Fig. 8*B*–*D*; *n* = 8 brains for each type; WT: *n* = 192; KO: *n* =204, *p = 0.0001^s^ for axon growth and *p = 0.0001^t^ for number of branches at 150, 200, 250, and 300 µm). During perinatal development, RGCs axons from both eyes connect with multiple target cells in the dLGN. These projections spread throughout the dLGN sharing common terminal space. Eye-specific segregation occurs during postnatal development (Godement et al., 1984). In the adult rodent, RGC axons occupy distinct eye-dependent non-overlapping regions of the dLGN. To assess the involvement of GPR55 in retinogeniculate development, we examined the projections to the dLGN of adult *gpr55^−/−^* and their WT littermates. Contralateral projections of *gpr55^−/−^* mice occupied a larger area than that of *gpr55^+/+^* mice (Fig. 8*E*). The contralateral and ipsilateral retinal projection images were quantified using a multithreshold method of analysis. These data indicate a significant overlap between contralateral and ipsilateral RGC projections in the dLGN of *gpr55^−/−^* mice [Fig. 8*F*; *n* = 7 brains (140 slices) for WT and *n* = 7 brains (140 slices) for KO; *t = 0.0234^u^; df = 38].

Compared to other rodents, hamsters have a shorter gestation period [hamsters (15.5 d), rats (21.5 d), and mice (18.5 d)]. Therefore, hamsters are born with a relatively premature neurovisual system at birth (Clancy et al., 2001). The embryonic development of the neurovisual system in these mammals (mouse and hamster) occurs at almost identical time points. For example, RGC generation starts at E9.5 for hamsters and E10.5 for mice, whereas the dLGN starts to develop at E10.5 for both models (Robinson and Dreher, 1990; Clancy et al., 2001). The RGC axons of hamster reach their thalamic and midbrain targets at (P3; Bhide and Frost, 1991). Taking advantage of this observation, intraocular injections were performed in hamsters at P1, to investigate the effects of GPR55 ligands during the early development of the visual system.

To assess the contribution of GPR55 ligands to the development of the retinal projections, the hamsters received intraocular injections of a GPR55 agonist or antagonist: LPI and CBD, respectively, at the date of birth. At P5, immunohistologic investigation revealed that interfering with GPR55 signaling had detrimental effects on RGC axon development. As indicated by a robust labeling of hamster retinal axons, LPI injection at the day of birth induced aberrant projections in the ipsilateral side of the SC (Fig. 8*G*). GPR55 pharmacologic agents modulated collateral projection length: compared with the control group, intraocular injection of LPI induced a significant increase in RGC axon growth and branch number in the DTN (Fig. 8*H*). Conversely, CBD decreased these parameters. Specifically, LPI injection induced a significant increase in RGC collateral length and branch number in the DTN, whereas these measures were significantly lower in the group treated with CBD compared with the vehicle group [vehicle: *n* = 84, 1µm; LPI: *n* = 25, 300 nm; CBD: *n* = 44, *p < 0.001^q^ for axon growth and *p < 0.0001^r^ for number of branches at 150, 200, 250, and 300 µm (LPI) and 100, 150, 200, and 250 µm (CBD); Fig. 8*I*,*J*]. Together, these observations demonstrate the important role played by GPR55 during the development of the retinogeniculate pathway.

## Discussion

In the present study, we show that GPR55 is expressed in the retina during the development of the visual pathway. GPR55 activation increased ERK 1/2 activity resulting in higher surface area and filopodia number of the growth cone. In addition, GPR55 agonist increased retinal axon growth, while a GPR55 antagonist decreased it. Interestingly, at high concentration, LPI can also activate the RhoA pathway, which decreases the GC surface area and the filopodia number, resulting in axon retraction. *In vivo*, at P3, the absence of the GPR55 causes a decrease of the axon branch number and length in the DTN. Accordingly, a decreased overlap between ipsilateral and contralateral projections compared to WT was expected in the adult mouse LGN. Interestingly, the opposite effect was observed which refers to a possible role of GPR55 in target innervation and refinement process. GPR55 activation with LPI in P5 hamster increased RGC projection length, branch number in the DTN and induced aberrant projections in the SC, whereas its blockade using CBD mimics the effect observed in the DTN of the *gpr55^-/-^* mouse. Together, these observations demonstrate that GPR55 plays an important role in axon growth and visual brain innervation. Furthermore, this receptor is crucial for proper development of the retinothalamic pathway.

### GPR55 expression in the retina

Previous studies reported ubiquitous distribution of *gpr55* mRNA in the CNS, with the following order of expression in mouse tissues: frontal cortex > striatum > hypothalamus > brain stem > cerebellum = hippocampus > spinal cord (Ryberg et al., 2007). It is also expressed in the caudate, putamen, dorsal root ganglion neurons, and differentiated PC12 cells (Lauckner et al., 2008; Obara et al., 2011; Sylantyev et al., 2013; Wu et al., 2013). In adult vervet monkey, GPR55 protein localization was reported strictly in the photoreceptor layer of the retina with most prominent staining in the inner segments in rod (Bouskila et al., 2013a). In our study, GPR55 protein is largely expressed in the adult mouse retina. Similar difference in the pattern of expression of cannabinoid receptor 2 protein was observed between adult vervet monkeys and adult rodents (Bouskila et al., 2013b; Cécyre et al., 2013). In fact, CB2R is present only in Müller cells in the adult vervet monkey retina (Bouskila et al., 2013b), whereas it is localized in cone and rod photoreceptors, horizontal cells, some amacrine cells, bipolar and ganglion cells in adult mouse retina (Cécyre et al., 2013). Similar distribution to the mouse was observed in the rat retina, with CB2R being localized in retinal pigmentary epithelium, inner photoreceptor segments, horizontal and amacrine cells, neurons in GCL, and fibers of the IPL (López et al., 2011). The difference in the protein expression could be attributed to the differences in the visual system between rodents and primates and the physiologic role played by the receptor in each animal model. Although the studies report the presence of the receptor in the adult CNS (Sawzdargo et al., 1999; Wu et al., 2013) and adult vervet monkey retina (Bouskila et al., 2013a), its expression in retina and retinal projections of rodents during development were unknown. In this study, we demonstrate that GPR55 is expressed in the developing hamster and mouse retina, axonal projections, their growth cones, and filopodia.

### Effects of the GPR55 ligands on the GC morphology and axon growth

In this study, pharmacologic activation or blockade of GPR55 modulated GC morphology and axon growth of RGCs. Accordingly, GPR55 agonists LPI and O-1602 increased retinal projection growth, induced an expansion in the surface area and augmented filopodia number of GCs. On the other hand, the GPR55 antagonist, CBD, decreased the growth of retinal projections and induced GC collapse. These data are in accordance with previous studies in which LPI has been found to activate GPR55 in DRGs (Lauckner et al., 2008), osteoclasts (Whyte et al., 2009), lymphoblastoid cells (Oka et al., 2010), cancer cell proliferation (Andradas et al., 2011), and hippocampal slices (Sylantyev et al., 2013). Knowing the proliferative and pro-migratory effects of LPI in cancer cell lines, we would expect a neurite elongation in PC12 cells (Soga et al., 2005; Hu et al., 2011; Piñeiro et al., 2011). According to the literature on LPI, most studies showed significant effects when LPI was used in concentration ranging from 0.1 to 10 µm, 1 µm being the most commonly used concentration (Oka et al., 2007; Anavi-Goffer et al., 2012; Rojo et al., 2012). Most of the studies reporting an effect of LPI on intracellular calcium mobilization, proliferation, channels’ activation and migration used lower concentrations, such as 1–3 µm (Oka et al., 2007; Henstridge et al., 2009; Pietr et al., 2009; Whyte et al., 2009; Piñeiro et al., 2011). Our results are in accordance with Balenga et al. (2011), defining LPI (3 µm) as a chemoattractive molecule for microglia. Another study showed a highly migratory effect of LPI (1 µm) on the metastatic MDA-MB231 breast cancer cell line, which expresses GPR55 (Monet et al., 2009). In addition, LPI stimulated cell elongation at the same concentration range (Ford et al., 2010). However, these findings challenge the results of neurite retraction produced by LPI via GPR55 in differentiated PC12 cells (Obara et al., 2011) or the absence of effect of LPI in spinal cord axons (Guy et al., 2015). The discrepancy could be explained by the fact that Obara et al., (2011) used LPI and CBD at concentrations 5 to 30 times higher than the ones used in the present study. At these high concentrations (≥10 µm), these GPR55 pharmacologic ligands could also act on nonspecific targets or activate alternative signaling pathways, such as RhoA/ROCK1 as shown in the present study. This hypothesis could explain the opposite effects seen between the two ranges of concentrations for the same GPR55 agonist, and help to illustrate the complexity of interpreting experiments with these lipids. Furthermore, GPR55 protein levels in PC12 and in primary neurons are different and may produce distinctive effects. PC12 cells are derived from a chromaffin cell tumor, thus are neuroendocrine-derived, and may be quite different from primary neurons. (Frassetto et al., 2006). Hence, the pharmacologic differences in the effect of LPI can be explained by dissimilarities in the phenotype of these two cell types. An additional difference that arises between PC12 cell lines and RGCs is the morphologic development that is regulated by diverse factors operating during different time periods (Coombs et al., 2007) and can explain the effect observed with LPI. The absence of effect of LPI on guidance of nociceptive afferent axons in the developing spinal cord (Guy et al., 2015) could be attributed to a difference in neuron subtypes. Nevertheless, these findings highlight the distinct mechanisms by which GPR55 modulates the development of various neuronal populations.

### Axon growth and GC morphology reorganization are mediated by GPR55 via the ERK1/2 or RhoA/ROCK1 pathways

Although several Gα subunits have been implicated in GPR55 signal initiation (Ryberg et al., 2007; Lauckner et al., 2008; Henstridge et al., 2011), it appears in our study that stimulation of GPR55 results in the activation of the MAPK pathway. To characterize the mechanism by which GPR55 modulates growth cone morphology and axon growth, we examined the ERK1/2 pathway. We demonstrate that at 1 µm, the GPR55 agonist, LPI, increases ERK1/2 phosphorylation. This is in accordance with an increasing number of studies showing that LPI-stimulated GPR55 activates ERK1/2 (Oka et al., 2007; Kapur et al., 2009; Pietr et al., 2009; Whyte et al., 2009; Andradas et al., 2011; Pineiro et al., 2011). In addition, our findings illustrate that at a higher concentration; this GPR55 agonist increases RhoA/ROCK1 activity and highlight additional signaling pathways associated with GPR55. A consensus among the articles published on GPR55 reported the involvement of the actin cytoskeleton and the activation of RhoA (Lauckner et al., 2008; Kapur et al., 2009; Henstridge et al., 2010). This dual action of LPI on ERK and RhoA/ROCK1 pathways is concentration dependent. Downstream signaling pathways of GPR55 remain controversial and further studies are needed to characterize all the mechanisms implicated.

### Effects of GPR55 ligands on RGC turning

As the neurovisual system is established, axons travel relatively long distances guided by the concerted action of attractive and repulsive cues in a complex environment to reach their target. Located at the axonal tip, the GC is a highly motile structure detecting directional signals in the environment. Guidance cues, notably members of the netrin, semaphorin, ephrin, slit families, cell-adhesion molecules, morphogens, and growth factors, modulate the behavior and growth of axons (Dupin et al., 2013). GPR55, like CB1R and CB2R, could be another modulation mechanism of axon guidance (Argaw et al., 2011; Duff et al., 2013). GPR55 activity at the GC modulates retinal axon navigation; GCs are attracted in the presence of LPI microgradient, whereas CBD induced GC collapse and retraction. Based on these results, GPR55 plays a modulatory role in axon navigation by modifying the morphology and the behavior of the GC. These results show, for the first time, a role for GPR55 in axon guidance *in vitro*, and are in accordance with the literature of LPI effect on signaling, migration, and growth (Oka et al., 2007, 2010; Lauckner et al., 2008; Henstridge et al., 2009; Pietr et al., 2009; Whyte et al., 2009; Pineiro et al., 2011; Sylantyev et al., 2013), whereas the opposite effect was observed with CBD (Ryberg et al., 2007; Pertwee, 2007; Ross, 2009; Whyte et al., 2009; Balenga et al., 2011; Kallendrusch et al., 2013; Sylantyev et al., 2013).

### GPR55 affects target selection during development *in vivo*

LPI can be generated by the action of a phospholipase PLA2 that catalyzes the hydrolysis of an acyl group from phosphatidylinositol (PI; Grzelczyk and Gendaszewska-Darmach, 2013). Similarly, PLA1 can be also involved in the formation of LPI (Yamashita et al., 2013). In 2007, LPI was described as the potential endogenous agonist of GPR55 (Oka et al., 2007) and was found present in different ranges in normal human cells (ie, platelets (Billah and Lapetina, 1982), peripheral blood neutrophils (Smith and Waite, 1992), various cancer cell lines (Xiao et al., 2000, 2001; Xu et al., 2001; Ford et al., 2010; Oka et al., 2010; Andradas et al., 2011; Pineiro et al., 2011; Cantarella et al., 2011), endothelial cells (Bondarenko et al., 2010), animal cells [ie, mouse fibroblasts (Hong and Deykin, 1981), macrophages (Zoeller et al., 1987), and rat brain cells (Oka et al., 2009)]. The concentration of LPI varies from a tissue to another (37.5 nm per gram of tissue in rat brain, 2.5 µm in mouse serum, and 1.5 µm in samples of human plasma; Grzelczyk and Gendaszewska-Darmach, 2013).

During visual system development, RGC axons travel long distances to connect to their specific targets. Many guidance cues modulate their navigation and target recognition; GPR55 and its endogenous ligands could represent one set of cues. Indeed, our *in vivo* data show that pharmacologic manipulation of GPR55 signaling affects retinal projection growth and navigation. We showed that a single intraocular injection of LPI leads to the emergence of aberrant ipsilateral RGC projections in the SC. Indeed, LPI injection increased branching or stabilized ipsilateral projections that would have normally retracted. Moreover, we report an increase in the length of retinal projections and in the number of axons in the DTN following treatment with LPI and a decrease with CBD. In addition, we noticed the presence of aberrant ipsilateral RGC projections in the SC in the postnatal *gpr55^-/-^* mice. The increase in the branching observed in the *gpr55^-/-^* mice could be explained by a stabilization of the ipsilateral projections that would have normally retracted. In addition, our data show that genetic interference with the GPR55 activity profoundly affects retinal projection development and target selection. Accordingly, the important role played by GPR55 during RGC axon growth and refinement is demonstrated by the relative lack of eye-specific segregation of retinal projections in *gpr55^-/-^* postnatal and adult mice. We interpreted this as a deficit in eye-specific segregation of retinal projections. In WT animals, this process could be influenced by GPR55 endogenous activity at the retina and/or directly at the axon terminal. It is possible that the absence of GPR55 could influence retinal spontaneous activity, which is necessary for segregation and maintenance of specific inputs to the dLGN (Chapman, 2000) thus modifying the segregation outcome. Deficiency in eye-specific segregation might also occur as a result of the absence of functional GPR55 directly at the dLGN. In summary, modulation of GPR55 activity strongly affects retinal projection development and target selection. These observations are in accordance with previous studies showing the role of GPR55 in motility, migration, orientation, and polarization of different types of human cells, such as breast cancer cells (Ford et al., 2010; Andradas et al., 2011) and myenteric neurons in mouse and human colon (Li et al., 2013).

In conclusion, the present study shows for the first time, *in vitro* and *in vivo*, that GPR55 and its ligands are involved in axon growth and in projection refinement at their midbrain targets. In addition, it pinpoints the signaling pathways that mediate their effects. The identification of the mediators implicated in these mechanisms is a valuable venue for developing new therapeutic agents aiming at the regeneration and repair of the CNS.

## Synthesis

The decision was a result of the Reviewing Editor Esther Stoeckli and the peer reviewers coming together and discussing their recommendations until a consensus was reached. A fact-based synthesis statement explaining their decision and outlining what is needed to prepare a revision is listed below.

Both reviewers acknowledge that the additional round of revisions has improved the manuscript and are now in favor of publishing the manuscript.
